# Deep learning and ultrasound feature fusion model predicts the malignancy of complex cystic and solid breast nodules with color Doppler images

**DOI:** 10.1038/s41598-023-37319-2

**Published:** 2023-06-28

**Authors:** Han Liu, Chun-Jie Hou, Jing-Lan Tang, Li-Tao Sun, Ke-Feng Lu, Ying Liu, Pei Du

**Affiliations:** 1 Cancer Center, Department of Ultrasound Medicine, Zhejiang Provincial People’s Hospital (Affiliated People’s Hospital), Hangzhou Medical College, No. 158 Shangtang Road, Hangzhou, 310011 Zhejiang China; 2 Key Laboratory for Diagnosis and Treatment of Upper Limb Edema and Stasis of Breast Cancer, Zhejiang Provincial People’s Hospital (Affiliated People’s Hospital), Hangzhou Medical College, Hangzhou, 310011 Zhejiang China

**Keywords:** Breast cancer, Computational models, Network models, High-throughput screening, Image processing

## Abstract

This study aimed to evaluate the performance of traditional-deep learning combination model based on Doppler ultrasound for diagnosing malignant complex cystic and solid breast nodules. A conventional statistical prediction model based on the ultrasound features and basic clinical information was established. A deep learning prediction model was used to train the training group images and derive the deep learning prediction model. The two models were validated, and their accuracy rates were compared using the data and images of the test group, respectively. A logistic regression method was used to combine the two models to derive a combination diagnostic model and validate it in the test group. The diagnostic performance of each model was represented by the receiver operating characteristic curve and the area under the curve. In the test cohort, the diagnostic efficacy of the deep learning model was better than traditional statistical model, and the combined diagnostic model was better and outperformed the other two models (combination model vs traditional statistical model: AUC: 0.95 > 0.70, P = 0.001; combination model vs deep learning model: AUC: 0.95 > 0.87, P = 0.04). A combination model based on deep learning and ultrasound features has good diagnostic value.

## Introduction

As breast cancer is increasingly diagnosed in women, the incidence of breast cancer continues to increase, and compared with the decreasing mortality rate of lung cancer, the mortality rate of breast cancer remains high in female patients, which may be due to insufficient early detection and treatment^1^. Ultrasound is a quick, easy, non-invasive and radiation-free diagnostic modality that is more suitable for breast cancer screening than other diagnostic imaging modalities^2^. In breast ultrasonography, changes in shape, orientation, margins, echo patterns, posterior acoustic shadows, and blood flow (BF) are included in the breast imaging reporting and data system (BI-RADS) dictionary prepared by the American College of Radiology (ACR) as important features in the evaluation of breast cancer^3^. However, the ultrasound features of solid lesions do not apply to complex cystic-solid nodules (C-SNs) (defined in the dictionary as a combination of cystic and solid components/cystic lesions with thick walls and thick separations), and the dictionary features do not allow for a more accurate determination of the benignity or malignancy of complex lesions^4–6^. In addition, there is a risk of metastasis due to leakage of cystic fluid when percutaneous core needle biopsy or incomplete surgical excision is performed on malignant C-SNs. Therefore, a non-invasive and accurate diagnosis of C-SNs will promote a more rational treatment plan and decrease the rate of positive margins and re-excision.

Previous studies have shown that nodules size, blood flow characteristics, nodule margins, calcification and other ultrasound features correlate with the malignancy of complex C-SNs^7^. Since breast cancer tumor nodes depend on blood supply, rich blood supply is usually established in the nodes and these can be well detected by ultrasound color Doppler imaging, so color Doppler blood flow pattern is an important indicator for determining breast cancer^8,9^

Deep learning (DL) as an image recognition and interpretation model has been widely applied to the automatic recognition of medical images with a high accuracy rate^10,11^. Through the investigation of ultrasound DL applications, it was found that DL methods can use the exponentially increasing computational power of graphics processing units to identify abstract and complex imaging features without the region of interest(ROI) outlining, improving the objectivity of data sources and bringing great opportunities and application prospects for ultrasound DL imaging histology^12,13^. Compared with machine learning which cannot accurately label the ROI of ultrasound images with color Doppler, the choice of DL method is more suitable. The accuracy of breast cancer diagnosis has been significantly enhanced by the continuous advancement of DL and the frequent updating of models, rendering DL methods the most extensively utilized and precise models for breast cancer detection^14^. Our study establishes and validates a novel combined model (CM) that integrates DL, clinical data, and ultrasound features and then compare the accuracy of the CM, the DL model and the traditional model in the diagnosis of malignant complex C-SNs.

## Results

### Baseline characteristics

**Figure 1:**
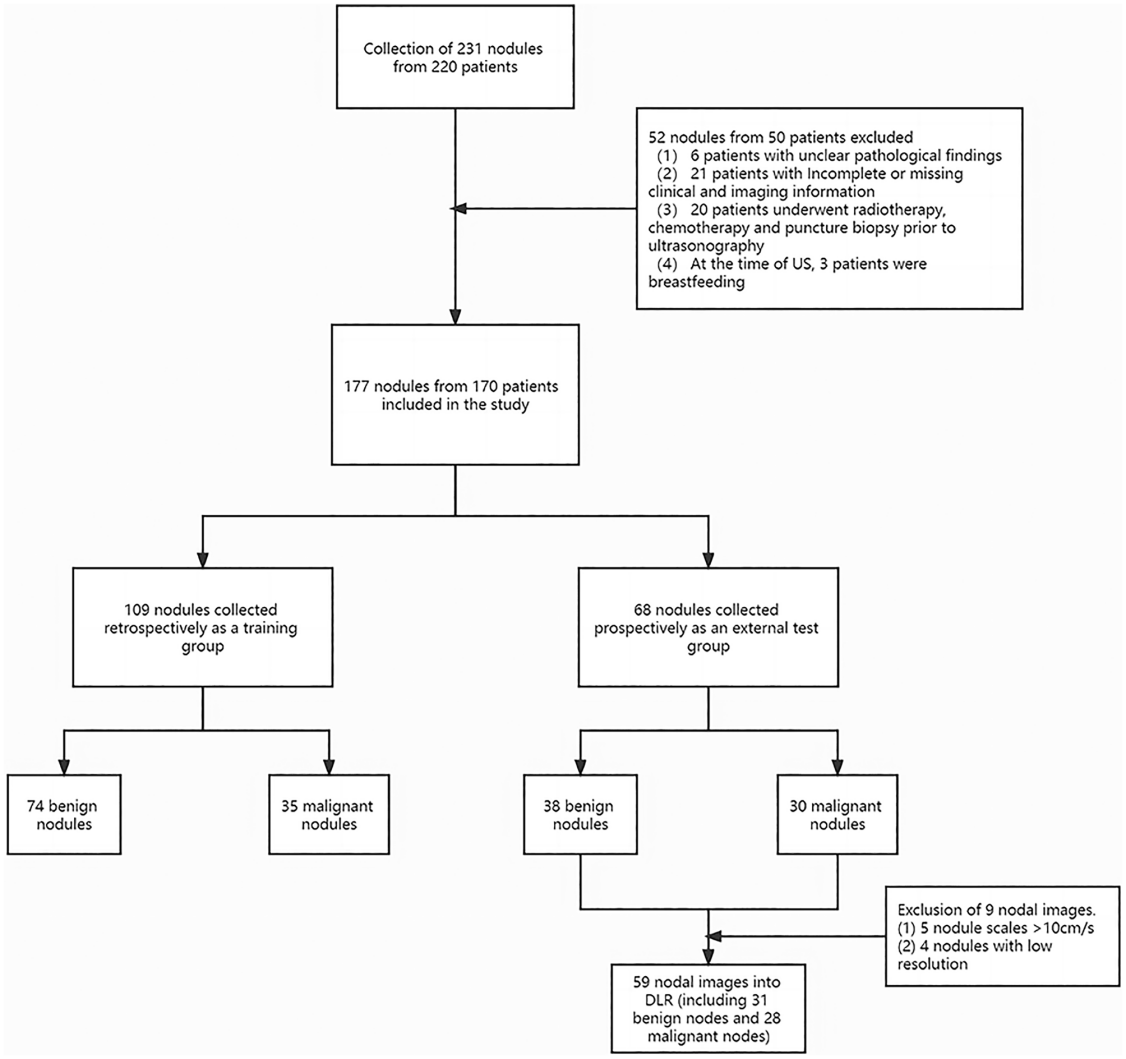
Data collection flowchart.

A total of 177 nodes from 170 patients were analyzed in this study, of which a total of 209 color Doppler images were collected (a node may appear in a different plane). The 9 test group nodes were eliminated from the group because the images did not meet the requirements, so the total data was 168 nodes, of which 109 were for the training group and 59 were for the testing group (Fig. 1). Baseline information is shown in Table 1. The ultrasound features in the baseline table were evaluated by two breast ultrasound experts, and the results were evaluated using the Kappa coincidence test, and the Kappa was 0.77 (P < 0.001), indicating high coincidence between the two experts in assessing the baseline data.

**Table 1:** Patient and ultrasonography baseline characteristics.

Characteristic	Total n percentile (%) *N* = 168	Training group *N* = 109 (64.9%)	Testing group *N* = 59 (35.1%)	Significance (*p*)
Age (year)	46.2 ± 12.1	46.9 ± 12.5	44.9 ± 11.3	0.320
Height (cm)	159.4 ± 5.7	159.2 ± 5.8	159.6 ± 5.5	0.657
Weight (kg)	59.2 ± 7.6	59.6 ± 7.8	58.4 ± 7.1	0.308
Transverse diameter (mm)	19.2 ± 15.1	20.7 ± 16.0	16.4 ± 13.2	0.075
Longitudinal diameter (mm)	11.7 ± 10.5	12.8 ± 11.2	9.4 ± 8.7	0.043*
Pathological results: *n* (%)				0.648
Benign	112 (66.7%)	74 (67.9%)	38 (64.4%)	
Malignant	56 (33.3%)	35 (32.1%)	21 (35.6%)	
Lactation history: *n* (%)				0.001*
F	47 (28.0%)	11 (10.1%)	36 (61.0%)	
T	121 (71.0%)	98 (89.9%)	23 (39.0%)	
History of menopause: *n* (%)				0.001*
F	84 (50.0%)	73 (67.0%)	11 (18.6%)	
T	84 (50.0%)	36 (33.0%)	48 (81.4%)	
Family medical history: *n* (%)				0.017*
F	158 (94.0%)	106 (97.2%)	52 (88.1%)	
T	10 (6.0%)	3 (2.8%)	7 (11.9%)	
Margin: *n* (%)				0.401
Clear	104 (61.9%)	70 (64.2%)	34 (57.6%)	
Unclear	64 (38.1%)	39 (35.8%)	25 (42.4%)	
Lesion shape: *n* (%)				0.823
Regular	76 (45.2%)	50 (45.9%)	26 (44.1%)	
Irregular	92 (54.8%)	59 (54.1%)	33 (55.9%)	
Cystic fluid transmission: *n* (%)				0.796
Clear	116 (69.0%)	76 (69.7%)	40 (67.7%)	
Poor	52 (31.0%)	33 (30.3%)	19 (32.3%)	
BF: *n* (%)				0.107
Absence	113 (67.3%)	78 (71.6%)	35 (59.3%)	
Abundant	55(32.7%)	31 (28.4%)	24 (40.7%)	

*BF* blood flow distribution.

*Indicates statistically significant difference.

### Traditional logistic regression model

**Figure 2:**
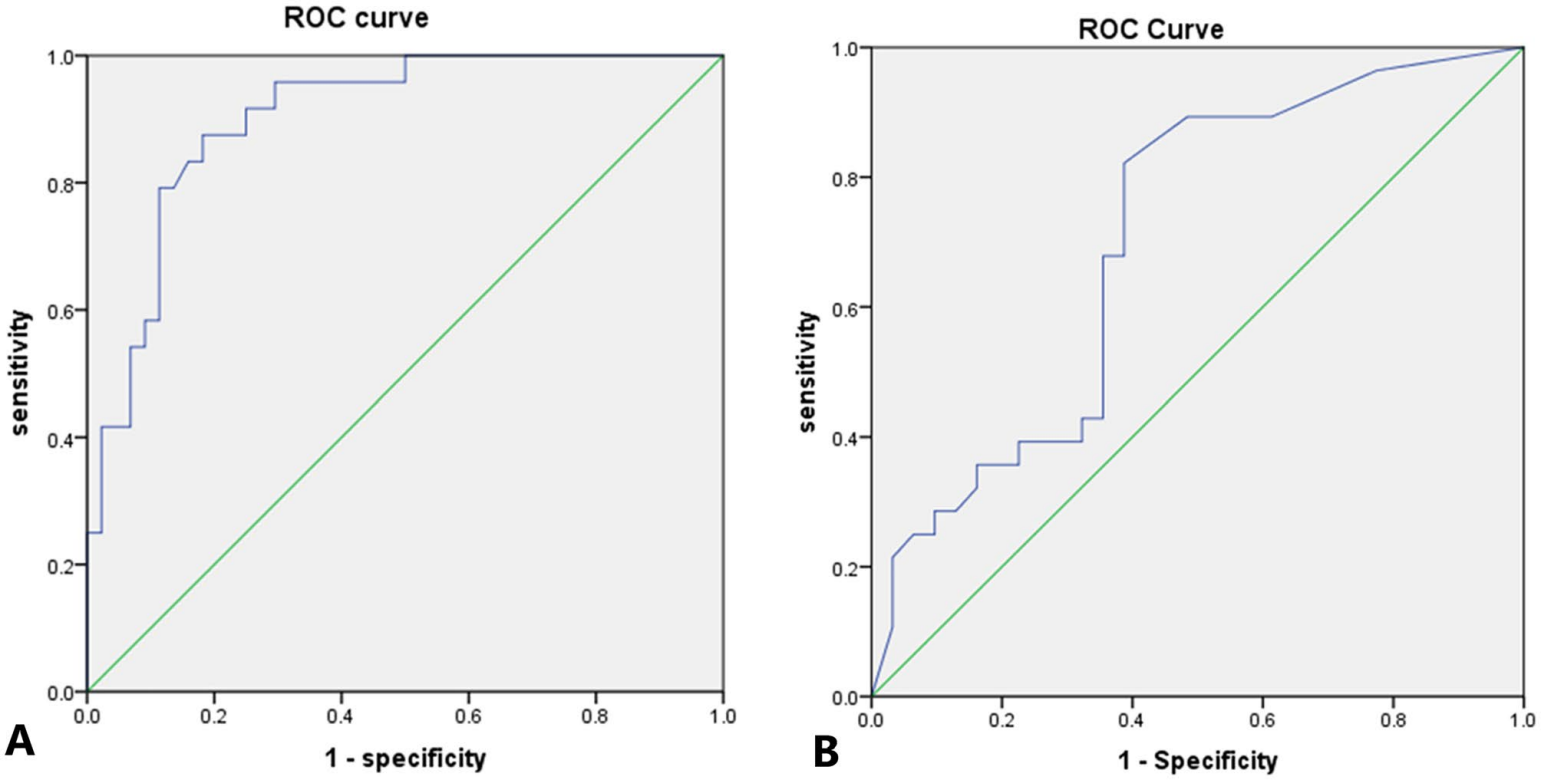
Receiver operating characteristic curve (ROC) curves for traditional statistical models. (**A**) Training group. (**B**) Testing group.

In the training group, there were statistically significant differences between the benign and malignant groups in the comparison of clinical and ultrasound characteristics, including age, height, size of nodules, Lactation history, history of menopause, Margin, Lesion shape, Distribution of cyst-solid components, Cystic fluid transmission, Presence of sponge-like structures/capsules and blood flow distribution (*P* < 0.05).The above characteristics were used as independent variables and benign/malignant as dependent variables into a training group logistic regression to establish a multiple stepwise logistic regression equation. After screening, BF (odds ratio (OR): 7.16; 95% confidence interval CI 1.17–43.67; P = 0.03), cystic fluid transmission (OR: 9.40; 95% CI 1.55–56.77; P = 0.02), longitudinal diameter (OR: 1.49; 95% CI 1.23–1.81; P < 0.01), and age (OR: 1.11; 95% CI 1.00–1.23; P = 0.03) were the independent predictors of malignant nodules. The accuracy of the model was tested with the test group data and the ROC curve was plotted and the results were calculated as AUC = 0.70, cut off value of 0.035, sensitivity of 0.66, specificity of 0.79, positive predictive value of 0.82 and negative predictive value of 0.61(Table 2 and Fig. 2).

**Table 2:** Validation results of each model in the testing groups.

Models	AUC	Sensitivity	Specificity	Positive predictive value	Negative predictive value	95% CI
Model_Traditional_	0.70	0.66	0.79	0.82	0.61	0.57–0.84
Model_DL_	0.87	0.75	0.85	0.86	0.74	0.76–0.97
Model_Combination_	0.95	0.96	0.88	0.86	0.96	0.88–1.00

*DL* deep learning, *AUC* area under curve, *CI* confidence interval.

### DL model

**Figure 3:**
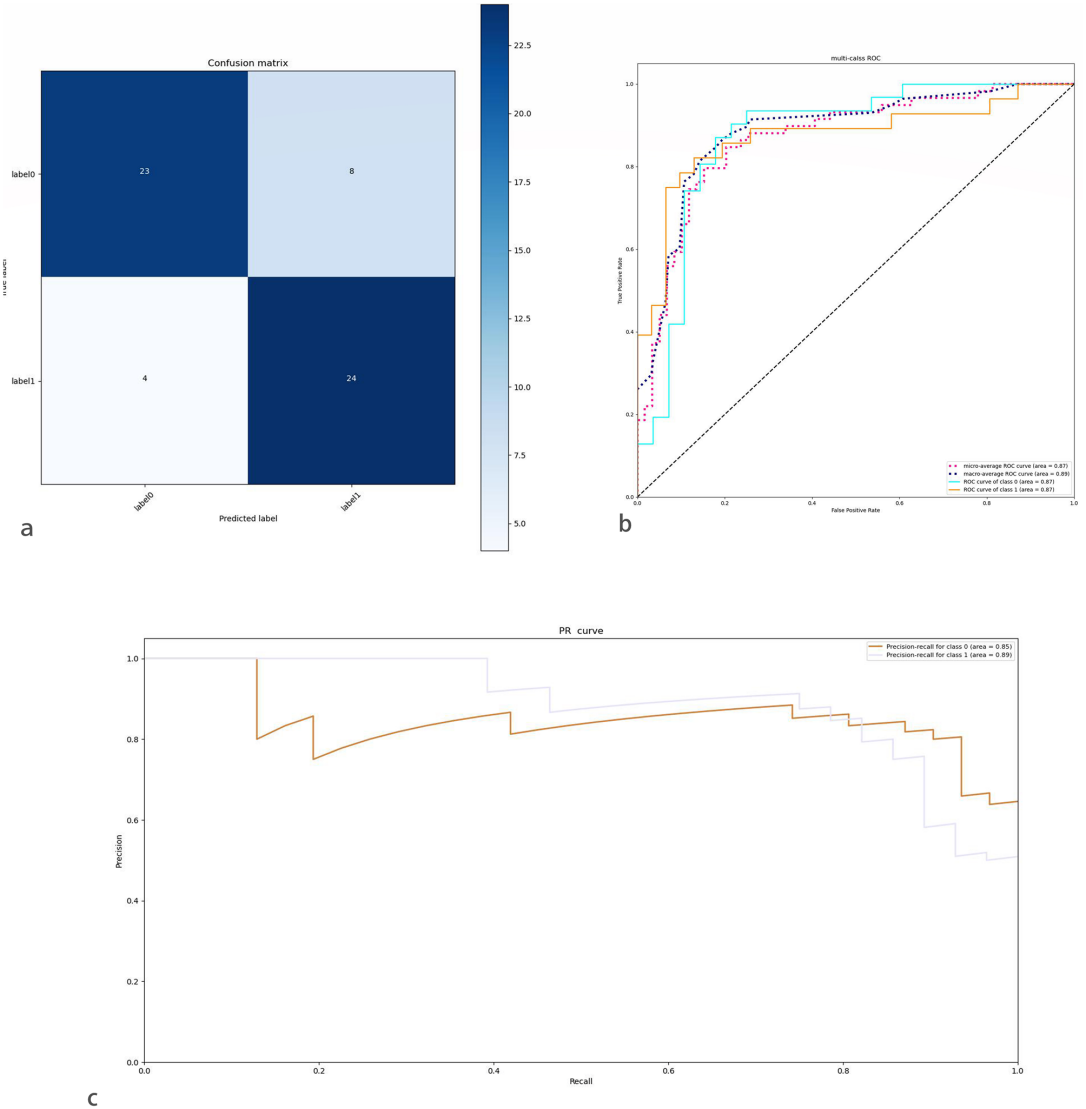
Visualization of DLR’s diagnostic results in the testing group. (**a**) Confusion matrix for diagnostic results. Label 0 = benign; Label 1 = Malignant (**b**) ROC curve of the testing group, the AUC is 0.87. ROC curve of Class 0: ROC curves based on negative predictive values; ROC curve of Class 1: ROC curves based on positive predictive values, micro/macro-average ROC curve: Summary curve of multiple roc curves (Area under the curve for multiple ROC curves > 0.87). (**c**) PR curves show that the deep learning model is more effective (maximum area under the curve > 0.89).

In this study, the DL showed superior accuracy. In the training group, the DL achieved an accuracy rate of 82%. In the independent testing group, the AUC was 0.87 (micro-average ROC curve), the model sensitivity was 0.75, the specificity was 0.85, the positive predictive value was 0.86, and the negative predictive value was 0.74. The confusion matrix and PR curves are plotted, and according to the PR curves, the sample has a well positive and negative equilibrium, and the model has a high applicability. The results are shown in Table 2 and Fig. 3.

### Clinical and ultrasound features combined DL

**Figure 4:**
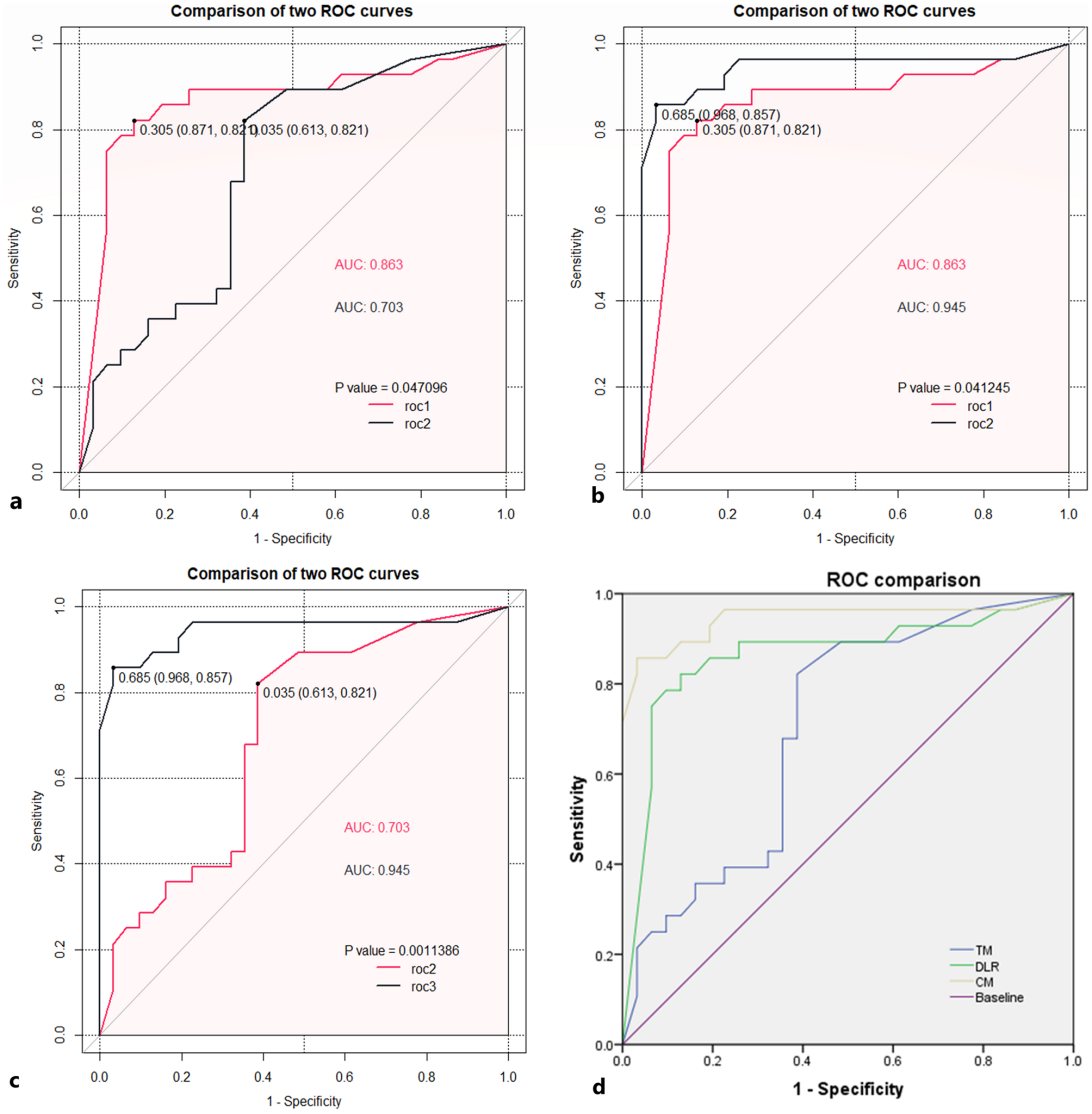
Comparison of the ROC curves between the three models. (**a**) ROC comparison between traditional statistical model and DLR model, roc1 DLR model; roc2 traditional statistical models(TM), *P* < 0.05. (**b**) ROC comparison between combination model (CM) and DLR model, roc1 DLR model; roc2 combination model (CM), *P* < 0.05. (**c**) ROC comparison between combination model (CM) and traditional statistical models (TM), roc2 traditional statistical models (TM); roc3 combination model (CM), *P* < 0.05. (**d**) Comparison of ROC curves and AUC between the three models.

The predicted values of the traditional clinical prediction model and the predicted values of each sample calculated by the DL model were used as independent variables, and the “glmnet” package in R was applied to create a new CM and calculate the AUC of 0.95. The model sensitivity was 0.96, specificity was 0.88, positive predictive value was 0.86, negative predictive value was 0.96, and the results are shown in Table 2 and Fig. 4.

### Comparative study of three models

In the test group, the AUC values of traditional statistical model, DL model, and CM were 0.70, 0.87, 0.95, respectively, and the differences between the three were statistically significant in two comparisons (*P* < 0.05). The model diagnostic efficacy of the CM > DL > traditional statistical model. The comparison between AUC values is shown in Table 2 and Fig. 4.

### The assisted diagnosis function of the CM

**Figure 5:**
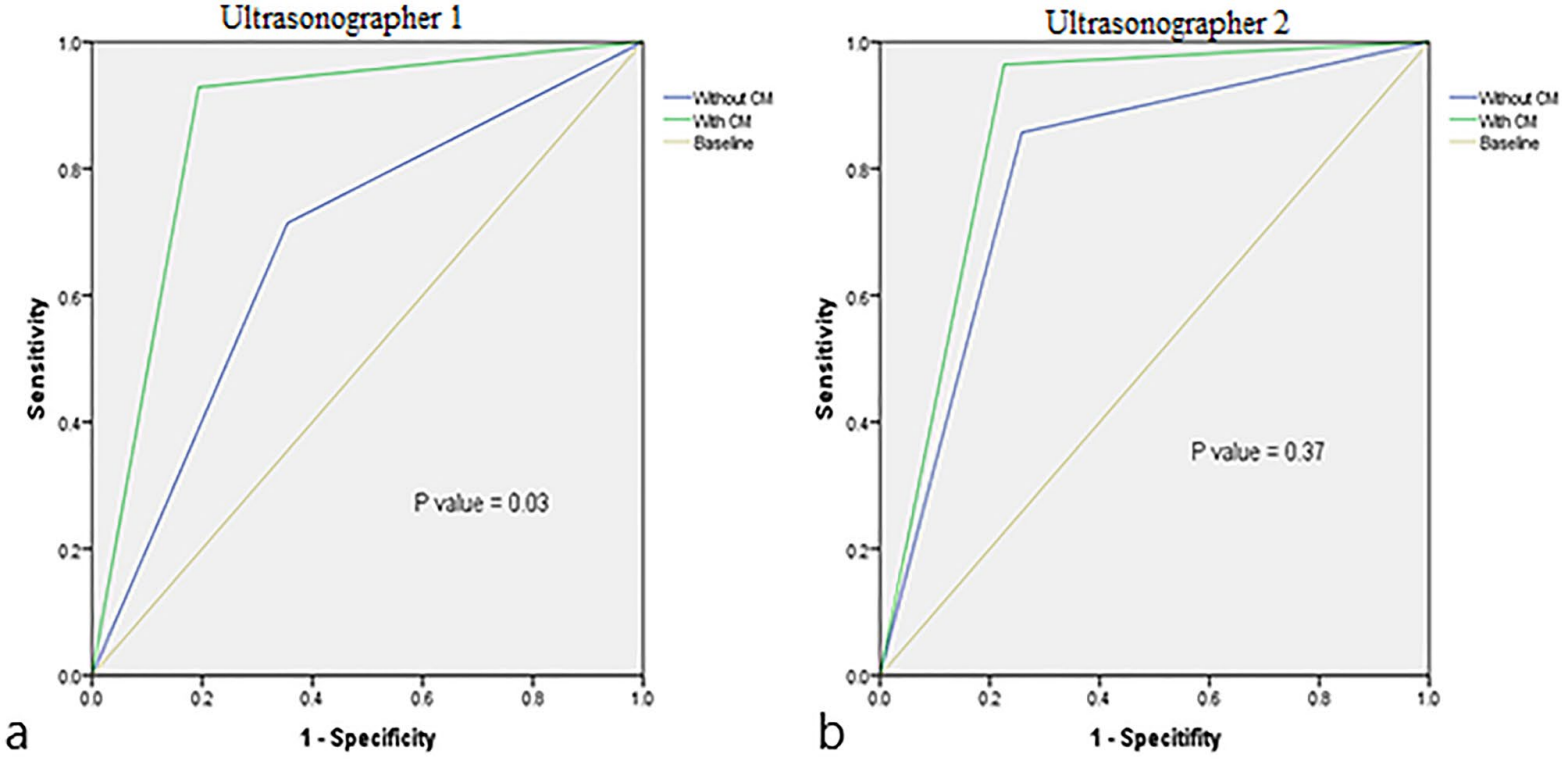
Comparison of the diagnostic performance of two experts before and after using the combination model (CM). (**a**) Ultrasonographer 1 has 3 years of experience in breast ultrasound diagnosis. AUC (with CM) vs. AUC (without CM), *P* = 0.03; (**b**) Ultrasonographer 2 has 5 years of experience in breast ultrasound diagnosis. AUC (with CM) vs. AUC (without CM), P = 0.37.

The diagnostic accuracy of ultrasonographer 1 (with 3 years of experience) was significantly improved after using the CM, and the difference was statistically significant in the comparison of AUC (AUC_with CM_: 0.87 vs. AUC_without CM_: 0.68, *P* = 0.03). The diagnostic accuracy of ultrasonographer 2 (with 5 years of experience) was not significantly improvement after using the model, and there was no significant difference in the comparison of AUC (AUC_with CM_: 0.86 vs. AUC_without CM_: 0.80, *P* = 0.37). The results were shown in Table 3 and Fig. 5.

**Table 3:** Comparison of the diagnostic performance of ultrasonographers with/without using the combination model.

Ultrasonographers	AUC	SE	Z value	Significance (*p*)
Ultrasonographer 1				
Without CM	0.68	0.07	− 2.20	0.03*
With CM	0.87	0.05		
Ultrasonographer 2				
Without CM	0.80	0.06	− 0.89	0.37
With CM	0.86	0.05		

*CM* combination model, *AUC* area under curve, *SE* standard errors.

*Indicates statistically significant difference.

## Discussion

Fatma Kulali et al.^15^found that complex C-SNs of the breast are usually predominantly benign nodules with pathology of fibroadenomatoid/fibrocystic changes and malignant nodules with pathology of intraductal papillary carcinomas, and cystic lesions are usually more common with benign lesions. However, C-SNs have a wide probability of malignancy in previous studies (20–40%)^16,17^. Although there have been more studies in the last decade to determine the benignity and malignancy of complex cystic nodules of the breast, there is still no consensus on the specific ultrasound features. HUILING XIANG et al.^18^ concluded that complex cystic solid nodules should be classified as more than four categories in BI-RADS, and biopsy or resection should be performed if necessary, and the clinic should give the necessary research and attention.

In addition, cystic thyroid nodules are considered one of the main causes of false-negative results on routine fine-needle aspiration biopsy, limiting the ability of aspiration biopsy to assess malignant nodules, and although there are no reports related to positive aspiration rates for complex C-SNs of the breast, the false-negative rate of aspiration biopsy of complex cystic nodules of the breast requires vigilance and concern^19^. Whether the rupture of the cystic component resulting from the incomplete removal of the lesion during surgery could lead to the spread of the cancer cells to the surrounding area also requires vigilance. It is of clinical relevance to the decision to puncture and to the choice of surgical procedure, if the malignancy of complex cystic nodules of the breast can be predicted preoperatively or before puncture.

In this study, age, longitudinal diameter, cystic fluid transmission, and blood flow distribution were found to be independent predictors of the benignity and malignancy of complex cystic nodules of the breast in a statistical model based on clinical and ultrasound characteristics. In a pathological study of three malignant complex C-SNs, YASUKO Mizushima et al. found that multiple structures within malignant tumors were susceptible to liquefaction necrosis due to the restricted tumor growth environment, and due to the rich blood supply to the tumor, they were prone to form a cystic result of hemorrhagic necrosis, which showed a poorly sonographically transmissive cystic component, which is consistent with the finding that cystic fluid transmissibility was the most significant predictor of malignant nodules in this study^20^. In addition, in a case report by Kyung Hee Ko et al.^21^, it was found that malignant breast cancer nodules showed short-term re-emergence of hemorrhage in the cystic wall after aspiration of the cystic component, which on the other hand proves that the hemorrhagic cystic component is a more specific ultrasound feature of malignant complex C-SNs. In this study, blood flow distribution and age as well as nodule size were also found to be more important predictors of malignant nodules, and the results were consistent with the findings of Ying Zhang et al.^22^. Worryingly, in this study, the AUC calculated by the conventional statistical model in the independent test group was only 0.70, yet in the training group the AUC value reached 0.92 (95% CI 0.88–0.99) and the model appeared to be overfitted. Therefore, we introduce DL to predict malignant complex C-SNs.

Artificial intelligence has shown high accuracy and AUC values (> 0.9) compared to traditional statistical models in previous breast nodule prediction models, with DL being more prominent^23–26^. Machine learning requires lesion outlining and requires a high level of operator skill and experience, while DL has a wider applicability than machine learning, as it does not require lesion outlining and can be used for image recognition with complex information^27,28^. In this study, we added color Doppler ultrasound images to DL and innovatively brought blood flow distribution characteristics into the DL to obtain good prediction results (AUC = 0.87). And through comparison, the diagnostic efficacy of DL models was found to be significantly better than that of traditional statistical models.

With advances in quantitative imaging analysis, radiomics has become an effective tool to guide personalized diagnostic and treatment decisions^29^. However, it is not objective and comprehensive to depend only on radiomics or even DL for disease diagnosis^30^. In previous studies, combined diagnostic models based on DL models fused with other data sources could significantly improve the diagnostic performance of the models^31,32^. Xueyi Zheng et al.^26^ found that clinical combined DL predicted lymph node metastasis in breast cancer showed better diagnostic results compared to a single DL diagnostic model through a study. In this study, traditional statistical models are linked in series with DL models through logistic regression to derive a CM, and such a model then includes acoustic features summarized by two experts, some relevant clinical data, and DL model features. In this study, it was found that the CM significantly improved the diagnosis of malignant C-SNs for junior ultrasonographers, however, there was no significant improvement in the diagnosis of senior ultrasonographers. The production of mucin and vascular substances by breast cancer results in specific image features that are often detected by experienced sonographers using color Doppler ultrasonography to visualize the blood supply. This may explain why the diagnostic ability of senior ultrasonographers was not enhanced by the model^33,34^.

The present study had some limitations. First, this study is a single-center study, and a multi-center study is needed to test and refine the model and improve the generalization ability of the model. Second, the sample size of this study was small and the number of cases with complex C-SNs in the breast was lacking due to the limitation of a single center, and future additions are needed to improve. Third, this study was limited by hardware conditions, only able to run Resnet50. VGG and intercept_V3 could not be supported, and the choice of the migration learning model was limited. In the future, this study can explore the association of different genotypes with complex C-SNs of the breast by substituting pathological typing and genetic markers into a DL model for radiomics-genomics studies.

Clinical and ultrasound features combined with DL model nodule reading color Doppler ultrasound images have high accuracy in predicting malignancy of complex cystic breast nodules, which can then provide a reference for malignancy determination of complex cystic breast nodules and provide effective suggestions for whether to intervene in clinical. We expect that subsequent multi-center validation will provide a higher level of evidence for clinical application and include more DL models for comparative studies.

## Methods

### Patients

A retrospective analysis of the data from the Zhejiang Provincial People’s Hospital in Hangzhou, Zhejiang, China was conducted and approved by the Zhejiang Provincial People’s Hospital Medical Ethics Committee, and all the methods were carried out in accordance with the principles of the Declaration of Helsinki. In this study, 155 nodules were collected from 148 patients described as complex C-SNs and ACR BI-RADS category 4a and above on the US reports from 2018 to 2021. Data and images collected in retrospective study as training group. In addition, this study prospectively collected data and pictures related to 76 nodules from 72 patients as a test group according to the requirements of the training group. The above data were obtained after surgical or percutaneous puncture and the corresponding pathological results were obtained. Written information was provided and informed consent was obtained from all subjects.

Inclusion and Exclusion Criteria 1. Inclusion Criteria: (a) All breast nodules had a BI-RADS classification of 4a and above; (b) All nodules were described as C-SNs in the ultrasound reports; (c) All nodes were operated for complete pathological results. 2. Exclude candidates: (a) A nodule with unclear pathological findings was found; (b) Incomplete or missing clinical and imaging information; (c) Radiotherapy, chemotherapy, and puncture biopsy were administered to patients before US examination; (d) At the time of US, the patient was breastfeeding.

After selection, 177 nodules from 170 patients were included in the study. 109 nodes from the ultrasound picture archiving and communication system (PACS) retrospective data were selected to the training group, including 74 benign nodes and 35 malignant nodes. A total of 68 nodes from prospective data were selected for the test group, including 38 benign nodes and 30 malignant nodes, of which only 59 images of nodes were selected for the DL test group due to some images not meeting the requirements. The details could be seen in Fig. 1. According to the ACR BI RADS classification, 121 (72%) nodes were classified as grade 4a, 23 nodes (14%) were classified as grade 4b, 15 nodes (9%) were classified as grade 4c, and 9 nodes (5%) were classified as grade 5.

### Clinical data and ultrasonographic feature collection

Clinical characteristics included age, height, weight, history of lactation, history of menopause, and family history. Among them, age, height, and weight were continuous variables, and lactation history, menopause history, and family history were categorical variables. The color ultrasound Doppler images were obtained from multiple ultrasonic diagnostic apparatus, including Philips Epic 5 ultrasound system (Philips Medical Systems, Bothell, WA, USA), Supersonic Aixplorer ultrasound system (Supersonic Imagine, Provence, France), Mindray Resona 7, and Mindray DC-8 (Mindray, Shenzhen, China). This study used a high-frequency line array probe with a center frequency ≥ 12 MHz, using only color Doppler mode (excluding other blood flow imaging modes such as energy Doppler) requiring clear color signals, reduced clutter and color spillover, with a scaler color of red/blue and a scale of 4–8 cm/s. Ultrasound features were extracted and judged by 2 breast ultrasound experts with over 5 years of experience in breast ultrasound diagnosis, respectively, and the features extracted included: Margin, Lesion shape, Distribution, Aspect Ratio, Distribution of cyst-solid components, Cystic fluid transmission, Cystic -solid intersection, Presence of sponge-like structures/capsules, Microcalcification, Internal vascularity/BF, and the above features were judged and subjected to dichotomous variations (negative as 0 and positive as 1). Any disagreement on the suitability of a trial for inclusion in the review was resolved by a consensus through discussion. In this study, the above characteristics of the training group were used as independent variables, and the benign and malignant outcomes were used as dependent variables. Multiple logistic regression was used to produce traditional statistical models and screen independent predictors, and the testing group was used to test model accuracy and calculate ROC curves.

### DL model

In this study, retrospective study data and images from January 2018-June 2021 were used as the training group (which included 25% randomized data as the validation group to guide the selection of hyperparameters), and prospectively collected study data and images from July 2021–August 2022 were used as the independent testing group. This study used Resnet50 as a pre-trained model.

In this study, the size of the input image was cropped to 224*224 pixels and normalized, the batch size was 64, and the training cycle was 30 rounds. To alleviate the effects of overfitting and sample imbalance, the training group images are scaled, randomly rotated, randomly cropped, contrast adjusted, hue adjusted and saturation adjusted using the data enhancement mode, and the number of training samples is significantly increased after data enhancement, with 1260 images in the malignant group and 1404 images in the benign group after expansion. Continuously update the model parameters by forward calculation and back propagation, and calculate the loss function. Validate the training model with independent testing group images, produce ROC curves and PR curves, and plot confusion matrix。

### Clinical and ultrasound features combined DL

**Figure 6:**
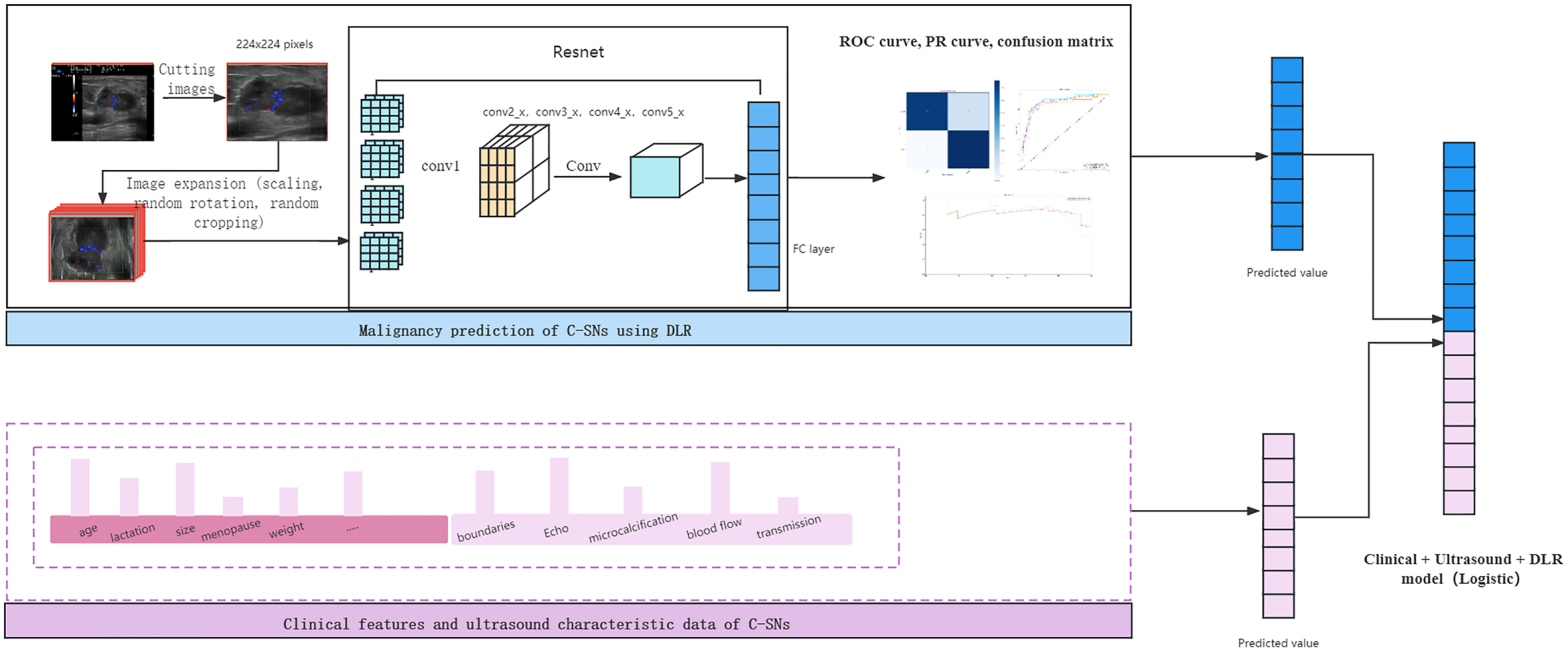
Flow chart of the two models and the process of model combination. *FC* fully connected layer, *ROC* receiver operating characteristic curve, *PR* precision recall, *DLR* deep learning radiomics.

In this study, two variables which were derived from the predicted values from the traditional statistical model and the predicted values from DL were brought into a new logistic regression equation as independent variables. We calculated the predicted values of the CM and plot the ROC curve. The areas under the ROC curves of these three models were compared to verify the accuracy of the models and to filter out the superior models. The model building process is shown in Fig. 6.

### Study of assistive functions of CM

In our study, we additionally selected 2 ultrasonographers with 3 and 5 years of experience in breast ultrasound diagnosis to identify benign and malignant nodules in the test group, with independent diagnosis in the first round and rediagnosis in combination with CM diagnosis in the second round. Their diagnostic accuracy with and without CM assistance was also compared.

### Statistical analysis

In this study, the data were classified into training and test groups, each of which was divided into benign and malignant groups, and baseline data on clinical and ultrasound characteristics were compared. Quantitative data were compared using *t* test or Mann–Whitney *U*, and qualitative data were compared using chi-square test. Consistency test of the results judged by two breast ultrasound experts using Kappa method. Traditional statistical models used multiple logistic regression equations. AUC values are used to compare the performance of the diagnostic capabilities of the three models. The Hanley & McNeil method was used to compare the diagnostic efficacy of the two ultrasonographers before and after using the CM. *P* values less than 0.05 in all statistical data were considered statistically significant. All statistical analyses were performed using SPSS (SPSS 23.0, SPSS Inc., Chicago, IL), R studio (based on R 4.2.1), Anaconda 3 (python 3.9).

## Data Availability

The datasets generated and analyzed during this study are not publicly available due to the requirements from the Ministry of Health of China of the guideline for the ethic review of biomedical research involving human subject (2016), but are available from the corresponding author on reasonable request.
